# Exploring key factors related to child well-being: a community-based participatory research together with children with migration background residing in socio-economically disadvantaged areas of Malmö, Sweden

**DOI:** 10.3389/fpubh.2025.1587678

**Published:** 2025-07-25

**Authors:** Louise Burenby Yxne, Rathi Ramji, Elisabeth Mangrio, Katarina Sjögren Forss, Therese Sterner, Margareta Rämgård

**Affiliations:** ^1^Department of Care Science, Faculty of Health and Society, Malmö University, Malmö, Sweden; ^2^Citizen Health, Malmö University, Malmö, Sweden; ^3^Malmö Institute for Migration Studies, Malmö University, Malmö, Sweden

**Keywords:** child well-being, Community-Based Participatory Research, participatory action research (PAR), migration, socioeconomically disadvantaged areas, socioecological model, Bronfenbrenner ecological systems theory

## Abstract

**Background:**

Children with migration background, living in socio-economically disadvantaged areas, are exposed to numerous risk that can negatively affect their well-being. Understanding which key factors build and enable well-being of children with these experiences are therefore essential to support and strengthen their positive development and possibilities to feel well. Studies that include children’s own perspectives and voices in initiatives that concerns them is however scarce, and there is an increased need for participation of children with diverse experiences and life situations in research and knowledge production. Therefore, the aim of this study was to, through a participatory process, explore and enhance the understanding of key factors in the social context that contribute to child well-being among children with a migration background.

**Methods:**

Thirty-one children, aged 9–12, from three disadvantaged areas in Malmö, Sweden, participated together with researchers in a Community-Based Participatory Research (CBPR) team, exploring factors related to their well-being in their social context. Multi-stage focus groups were held over a year (2023–2024), with different sessions facilitating dialog on the research topic. In total, 49 sessions took place, each on average duration of 1.5 h. The data were analyzed using inductive thematic analysis.

**Results:**

Four main themes, representing key factors related to child well-being, were identified: Enriching Leisure Time, Resourceful Places, Belonging to a Community and Welfare System and Rights. The themes covered structural aspects, such as school, healthcare and human rights, but also more personal dimensions, like close relations and the near environment, related to Bronfenbrenner’s ecological theory of human development.

**Conclusion:**

The dialogs with the children provided a deeper understanding and a holistic view of the perceptions of children with migration backgrounds residing in socio-economically disadvantaged areas regarding essential factors for their well-being. Future research should focus on developing strategies that ensure children’s access to the factors they have identified as fundamental to their well-being. Our study has further shown that it is pivotal to ensure children’s inclusion and participation in health promotion initiatives. These initiatives need to be contextually relevant and work toward building community capacity, to promote child well-being.

## 1 Introduction

This study addresses child well-being, which is fundamental to children’s physical, emotional, and cognitive development (1). Well-being is approached from a health-promoting perspective, focusing on key factors related to well-being among children with migration experiences living in socioeconomically disadvantaged areas, a group facing numerous risks that can adversely affect their well-being (2–6). In accordance with the Convention on the Rights of the Child, which was confirmed as official law in Sweden in 2020 (7), it is children’s right to be involved in decision-making processes that affect their lives. Moreover, including children’s perspectives and knowledge has shown to lead to more appropriate and successful initiatives, but also build children’s agency, empowerment and engagement in society (8, 9). Despite the increased intention to include child-perspectives, children’s participation in research is often limited only to parts of the research process (10). This Community-Based Participatory Research (CBPR) has been carried out in collaboration with children, who actively participated in data collection, analysis and distribution of results, to explore child well-being from children’s own perspective.

Traditional discourses on well-being of children orient from a time when children were seen as incapable of speaking or acting for themselves, with limited abilities to contribute to their own well-being actively and meaningfully (11). This perspective has changed, and children are more and more seen as capable agents who should be included in matters that concern them. Criticism is, however, directed at the domination of western perspectives within the discourse, as well as an insufficient exploration of children’s well-being from children’s own perspective (12). As insiders of a culture to which adults are outsiders, children provide unique insights essential for promoting their well-being, making their role in research vital (13). Moreover, given that the meaning of well-being is influenced by personal perceptions and experiences, it’s crucial to include children with diverse backgrounds in exploring its meaning (14).

*Child well-being* is a broad concept, and its meaning is both socially and culturally constructed. It can be divided into different domains ranging from emotional, social and physical well-being to economic well-being, indicating the width of the concept. It can further be divided into subjective well-being (personal perceptions and feelings) and objective well-being (objective and measurable data) (11). The following definition by the United Nations International Children Emergency Fund (UNICEF) is often used as a references in the field: “The true measure of a nation’s standing is how well it attends to its children – their health and safety, their material security, their education and socialization, and their sense of being loved, valued, and included in the families and societies in which they are born” (15). Aligned with this definition, current literature in the field recognizes that child well-being is a holistic, multidimensional, and dynamic concept (16) not least for migrant children (17).

As outlined, child well-being encompasses not only biological factors but also societal factors like economics, politics, societal systems, environmental factors (18, 19). Research shows that living in poor social and economic conditions negatively affects health and have a profound impact on children’s growth and development (18). The results of a national survey from 2021, showing that nearly 196,000 children live below the poverty line in Sweden, is therefore worrying. Most of these children reside in areas predominantly populated by migrant populations (20). As people with migration background have often gone through, or are currently experiencing, various situations and processes that negatively impact their health (21–23), children with a migration background living in socio-economically disadvantaged areas face multiple risks that can adversely affect their well-being. In addition to an increased risk of various physical and mental health problems among children with migration background (2, 3, 5), many also report feelings of powerlessness, low self-esteem, behavioral issues, feeling unsafe, low levels of life satisfaction, and loneliness (4, 5).

While there is extensive literature on health risks among children with migration background living in socioeconomic deprivation, research on their overall well-being seems sparse. Moreover, as described in a literature review by Bajo Marcos et al. (24), most studies within the field focus on adolescents rather than younger children, under 13 years old. A few studies investigating the influence of cultural conceptions of well-being among children with migration background do exists, but research employing a participatory action research approach in this context seems even more uncommon (24). In a related study, Smith (25) highlights similar research gaps, and argues for applying a strength-based approach to gain a more comprehensive understanding of well-being among children with migration background. Looking at factors related to well-being, instead of risks, implies a stance that focus on positive aspects rather than negative ones, which is the approach applied in our study.

While the circumstances for many children with migration background, living in socio-economic disadvantaged areas are difficult in different ways, most demonstrate strong resilience and develop positively despite the challenges they carry and the societal challenges they face (6, 12, 23). Identifying factors that contribute to children’s resilience and positive development is therefore pivotal. In this study, a strength-based approach (SBA) is applied to explore the promotive factors that support child well-being. The core premise of SBA is that people are experts in their own lives and individuals are seen as resourceful and capable. SBA is considered a respectful approach that acknowledges people’s abilities (26, 27). However, as children’s well-being is affected by the dynamic system within which they grow, including their family, friends, teachers, the neighborhood, societal norms and the political system, understanding child well-being also requires a broader societal perspective (28–30).

Since child well-being is shaped by the surrounding community and environment, incorporating the wider socioecological context is essential for effectively reaching children with promotive initiatives aimed at enhancing their well-being (25, 30). Community Health Promotion is one approach, acknowledging social, cultural, and environmental health influences, aiming not only at personal behavior change but also at broader societal structures (31, 32). Further, it is a recognized approach for reaching migrant populations in general (33), including children (25). Community Health Promotion, as demonstrated in our study, involves strategies aimed at improving the health and well-being of a geographically defined community, or a community of interest, by building capacity for community members to identify their own needs, develop solutions, and take action to address these needs. For children in a community, health promotion activities often include playful elements, such as active leisure time and play which are recognized as crucial for child well-being (34, 35).

A fundamental aspect of community health promotion is *empowerment*, the process through which individuals, organizations, and communities develop control and influence over matters that are important to them (36). Empowerment aligns with Paulo Freire’s critical pedagogy, and the concept of “Conscientization,” meaning development of critical awareness of one’s social reality. Through dialog, reflection and analysis, conscientization creates an understanding of oneself as a change agent with the ability to influence and transform (37–39). From this viewpoint, empowerment is not about attaining power to control or dominate others, but rather about having the ability to collaborate with others to create meaningful change (37). Applying *a participatory research approach*, using Bronfenbrenner’s ecological model (40) as a method, is one way of working toward empowerment.

### 1.1 Community-based participatory research

*Participatory Action Research* (PAR) was developed by the social psychologist Kurt Lewin (41). One version of PAR is CBPR, built to include and emphasize experiences and knowledge that might otherwise be overlooked in academic knowledge production. It is a collaborative effort that aims at creating social change from a bottom-up perspective in a community (42, 43). The CBPR approach is a collective research process, defined by Wallerstein et al. (44) as “collaborative efforts among community, academic, and other stakeholders who gather and use research and data to build on the strengths and priorities of the community for multilevel strategies to improve health and social equity”. CBPR is thus a research orientation that anchors the research in the community, and consider community-based knowledge equally important to the academic contributions (45). CBPR emphasizes an emancipatory stance, and in this study, children, activity leaders from the community center in three socio-economically disadvantaged areas, and researchers from the university collaborated on equal terms (46).

CBPR with children focuses on engaging them with the aim of understanding the issues that are relevant to them, analyze the data, report the findings and act upon them through participatory working methods (47). The children are recognized as experts and co-researchers, while the researchers become co-learners (13). Despite growing acknowledgment of the importance of including children in research and academic knowledge production, it is relatively uncommon to fully realizing children’s participation in research processes. Some of the challenges are the time-consuming process and the research environment that is often difficult to control, requiring great flexibility and close collaboration with community partners, as well as ethical challenges (8, 9, 48, 49). The risk of tokenism, referring to situations where children are included in activities or decision-making processes in a superficial or symbolic manner, without their input being genuinely considered or acted upon, is also considered high (10).

While health promotion environments in school-settings are not uncommon globally, they often have an agenda pre-determined by adults, mostly focusing on issues such as nutrition or physical activity (49–51). Working holistically and promoting children’s well-being in a broader sense in such initiatives, including children as active participants throughout the process, seems to be rarer. However, by investing in children’s well-being and including children in knowledge development, better support can be provided to them, laying the foundation for healthy lives and positive educational outcomes with effects that extend into adulthood (52). Therefore, the aim of this study was to explore and enhance child well-being in collaboration with children, through the following research question: What key factors in the social context contribute to the well-being of children with a migration background, as identified through a participatory process together with the children?

## 2 Method

While a supportive community and involvement in leisure activities in the local community holds great significance for children’s well-being (53), there are inequalities in the participation in such activates, as children with lower socioeconomic status are less active (54). The reasons are often the costs, the difficulties parents face in providing necessary support as many work shifts or irregular hours, accessibility and neighborhood safety (55, 56), but also that children and youth in higher socioeconomic groups are more often encouraged and socialized to participate in sports clubs and other organized activities (57). As a response to the lower participation in leisure time activities in socioeconomically disadvantaged areas in Malmö, the municipality has invested in an initiative called the AllActivity House (AAH) (58). The AAHs are community centers that offer free activities for all ages, every day of the week. Most activities are for children, during and after school, and all AAHs in Malmö follow the same, specific working model, based upon participation, relationship-building, and democratic processes. While the needs and wishes of the participants determine the activities at the AAH, the children are also included in other kinds of decision-making, such as recruitment processes of personnel (58). The AAHs are located at the school premises, which makes them familiar and easy to access for residents in the area, but they act independent of the school organizations. The AAHs conduct about 60–80 activities per day, and 2023, nearly 40,151 girls/women and 41,819 boys/men were regularly engaged in the activities (46).

This study was conducted at three out of four AAHs in three geographically distinct areas of Malmö (the third largest city in Sweden), where unemployment, poor housing, poor health, child poverty, and crime are prevalent. These areas are predominantly inhabited by immigrants and are classified as socially vulnerable (58, 59). Although located outside the city center, the distances between these areas and the city center of Malmö are relatively short. The three AAHs were selected through dialog with the AAHs, who considered the fourth one too newly opened to participate in the research project.

**Figure 1:**
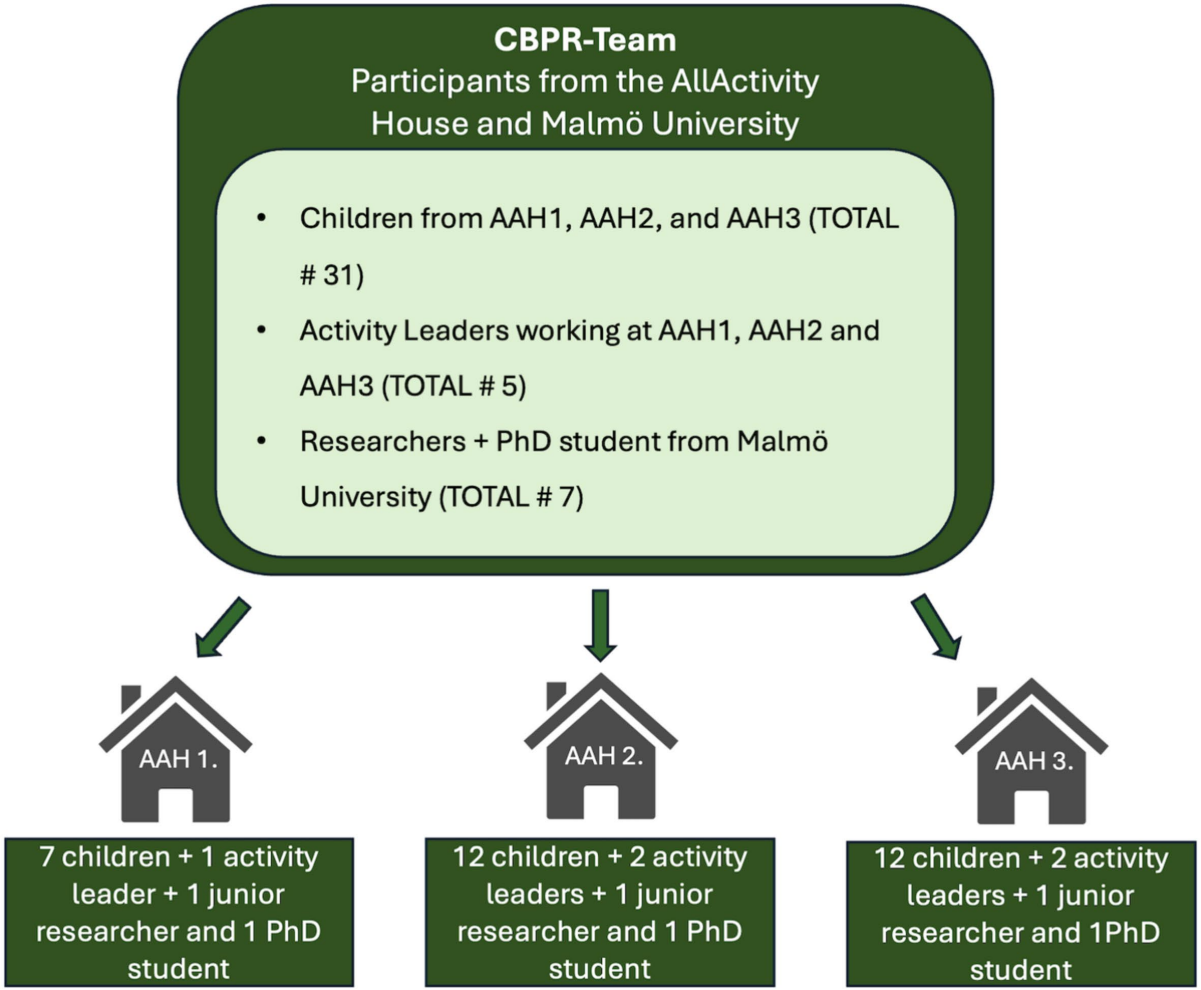
Structure of CBPR-team.

The research team in this study consisted of a group of junior and senior researchers, and one doctoral student. Working with CBPR was new to some, while others have long experience within the field. Most of the researchers in the team are born and raised in Sweden, while one has migration experiences. The CBPR-team included a total of 31 children, divided among the three different AAHs (see Table 1). There were two activity leaders participating in each group, but only one in the AAH with a smaller group of children (see Figure 1). The activity leaders, who work at the AAHs and meet the children weekly/daily, supported the sessions by ensuring that practical matters were handled and participated in weekly meetings to discuss progress with the first and second authors of this study. Primarily, the first and second authors met with the children in the AAHs on a weekly basis. The senior researchers who did not participate on a weekly basis in the AAHs still participated in regular meetings where the research was discussed and the data processed (46).

**Table 1:** List of child participants.

Characteristics	AAH1	AAH2	AAH3
Gender			
Boys	2	6	7
Girls	5	6	5
Age			
9–10 yrs	5	5	8
11–12 yrs	2	7	4
Total	7	12	12

The children participating were of mixed genders aged 9–12 who regularly go to the AAH. The children were first- or second-generation immigrants, most of them had a non-European background, many coming from the Middle East, with different religious background. An activity to learn research was started in the AAHs, facilitated by researchers together with activity leaders working at the AAH. The activity was initially open to all children aged 9–12 years from the respective AAH in three different neighborhoods.

While the overall participation remained stable, the number of children attending the sessions sometimes varied due to occasional conflicts with healthcare appointments, travel, or illness. Also, some of the children who participated in the first sessions later moved to other cities or even countries. Since multi-stage focus groups, as used in this study, allow for group dynamics to change, this was not a problem (60). No monetary compensation was provided, but milestones were celebrated with family parties and the children were always given a snack before and after each session.

### 2.1 Data collection and procedure

**Figure 2:**
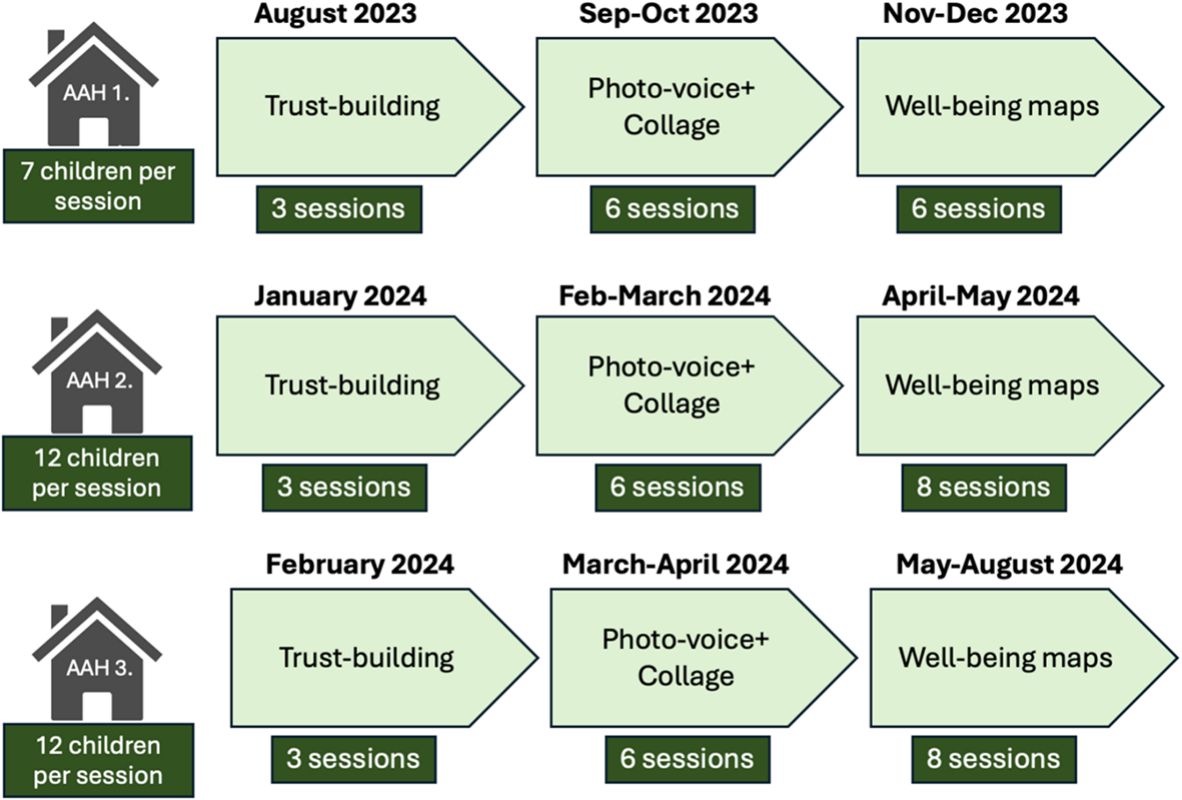
Chronological timeline of multi-stage focus group activities.

Data was collected using Multi-Stage Focus Groups (60). This method involves multiple focus-groups meetings over time, with various activities across these sessions to explore the research topic. Each session is conducted sequentially, building upon the insights and findings from the previous one. The method was used in this study to support the children’s ability to reflect through dialog and dive deeper into the research topic as their understanding increased over time. The children were active participants during data collection, parts of the analysis, and distribution of the results. The Multi-Stage Focus Groups in this study took place over a year (2023–2024), meeting on a weekly basis. To make the children feel comfortable participating, the research sessions took place in the premises of the AAHs. The research process started with a trust-building phase to build trust and engagement within the CBPR-teams. The CBPR-teams played games, got to know each other, and eventually the children got introduced to the research topic and what it means to be a child co-researcher. Thereafter, the team started to explore well-being through various activities. First, the photo-voice method was applied (61, 62) where the children were divided into two groups and equipped with mobile cameras. The first and second author followed the groups so that the children could take photos of things symbolizing well-being for them. The photos were printed ahead of the forthcoming session, and the children created collages with the pictures. Throughout the photo-taking and collage-making, which were team activities carried out together, the children engaged in dialogs to discuss and elaborate on the pictures, and what they signify for them in terms of well-being (see Figure 2) for overview of multi-stage focus group activities.

After the process of discussing the concept of well-being, key factors related to children’s well-being were explored. As children’s well-being is affected both by individual and structural factors (28), the research team took inspiration from Urie Bronfenbrenner’s ecological theory of human development. Bronfenbrenner’s ecological systems theory explains that human development is shaped by interactions within multiple layers of environment, from immediate family to broader cultural influences. The model consists of a set of systems, describing the different dimensions (29, 40, 63). The Microsystem, at the core of the model, includes the people and elements that have direct contact with the child, such as parents, siblings, teachers, and peers. These interactions directly influence the child’s life and development. The Mesosystem encompasses interactions between a child’s microsystems. Caregivers’ involvement in a child’s education and contact with the school and teachers, is an example. Lastly, the Macrosystem, referring to the broader socio-cultural context, includes cultural values, norms, legal frameworks, and economic systems in society.

In Bronfenbrenner’s traditional model, two more systems are included, called the Exosystem and the Chronosystem. The Exosystem includes larger social structures that the child does not directly participate in but still impact their lives. The Chronosystem is about time, considering changes that happens in a child’s life. These dimensions of the model were not deemed important for this study and were removed to not make it too complex (29, 40, 63).

**Figure 3:**
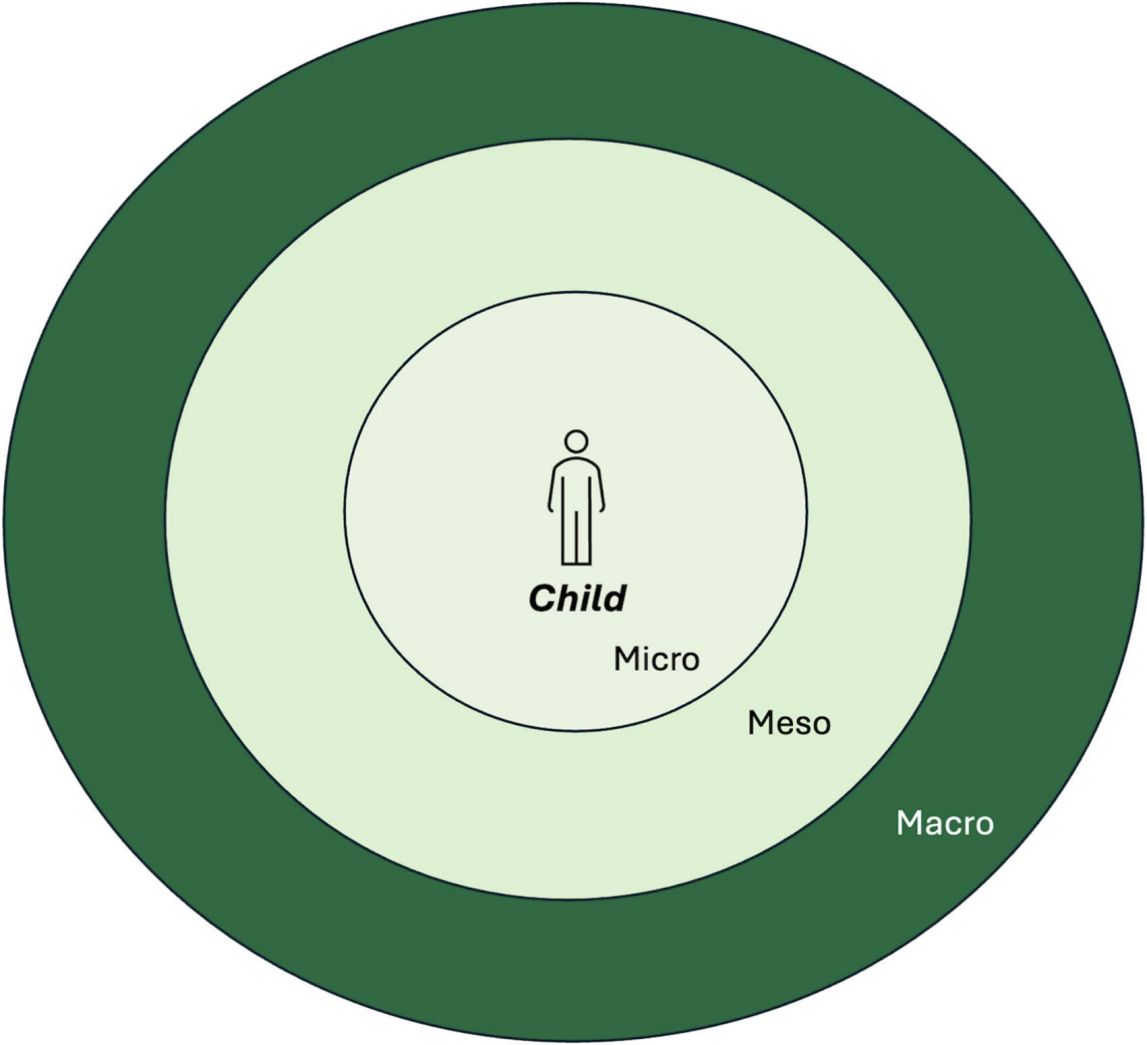
Visualization of the simplified version of Bronfenbrenner’s ecological theory of human development.

A visualization of Bronfenbrenner’s framework was developed in the form of a simplified and interactive hard-copy model (see Figure 3). Difficult terms were replaced, and overly complex aspects of the model were removed to be suitable for the children participating in the research. The model was namely used to let the children themselves define what factors they considered important to their well-being, and how great they considered their impact. The aim was to facilitate dialog on a structural level with the children.

**Figure 4:**
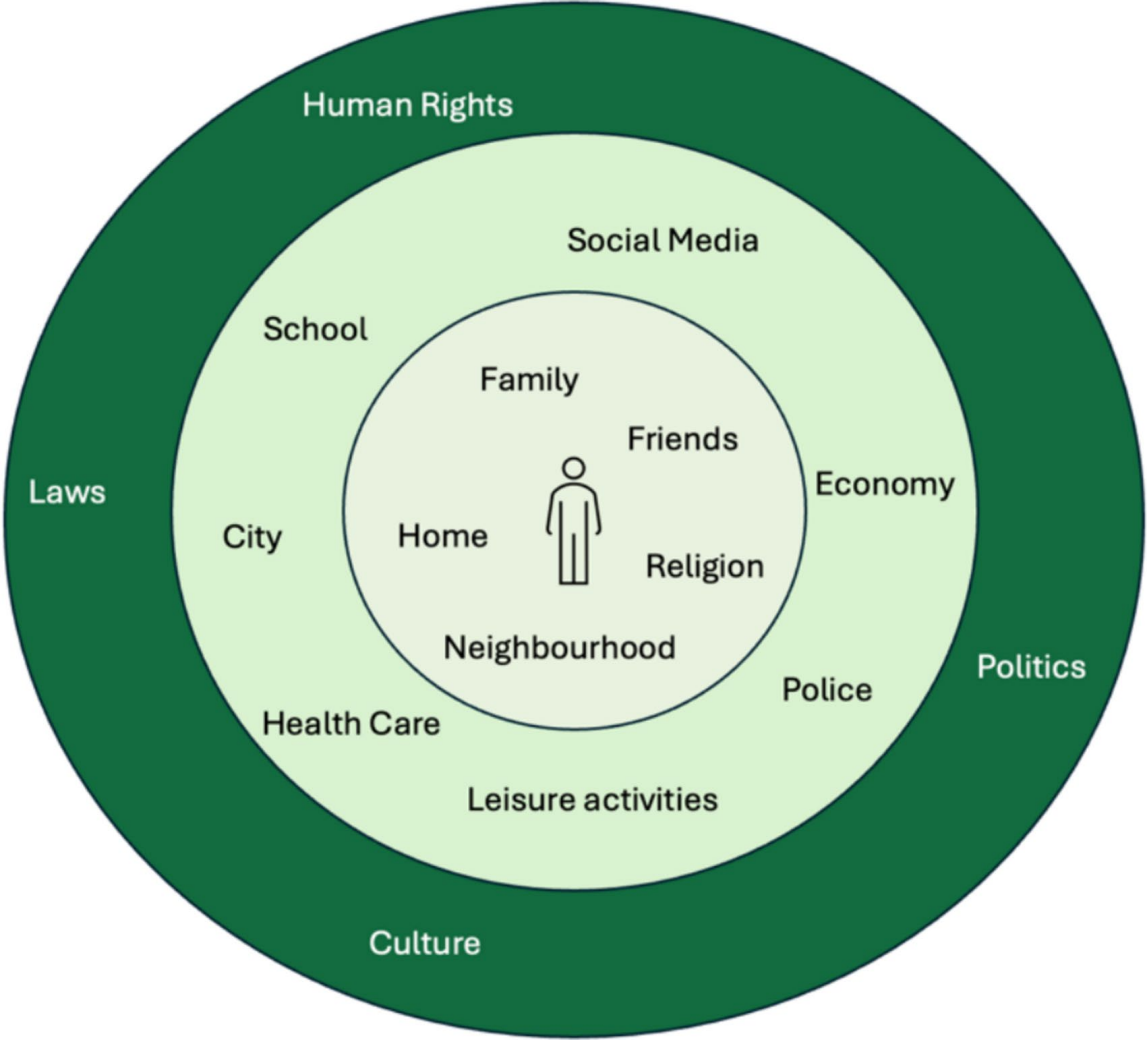
Example of how the children placed different factors related to their well-being.

Pictures that the children themselves had taken during the photo-voice sessions, as well as pictures symbolizing factors defined in Bronfenbrenner’s framework, were printed, and spread out on a table. One child at the time was welcomed to themselves choose as many pictures as they liked (or paint something new on a post-it if they missed anything), place them in the model and then describe *what* the thing on the picture meant to their well-being, *in what way*, and, *why* they placed it at a certain level in the model. The pictures placed in the middle were perceived as present in their daily life and directly related to their well-being. Figure 4 shows an example of how the children placed different factors related to their well-being. All children got to create their own “well-being map,” and an increased understanding of what factors they perceive as important was reached. All participants asked questions, engaged in the dialogs, and participated in the preliminary sorting and thematizing of factors related to well-being.

Flexibility was essential to meet the needs of the different groups of children and to ensure the sessions proceeded effectively. For example, if a child was angry, sad, or had difficulty concentrating, they were never excluded from the activity but were given time to rest or reflect, asked if something was wrong, and if anything could be done for them. Whenever a challenge was identified, various solutions were discussed among researchers and activity leaders, and the children were also consulted. Another strategy involved jointly deciding on “House Rules,” where the children themselves defined what was important to for creating a good environment during the sessions. This was done in the very beginning of the sessions after an initial phase of trust building.

### 2.2 Data analysis

**Figure 5:**
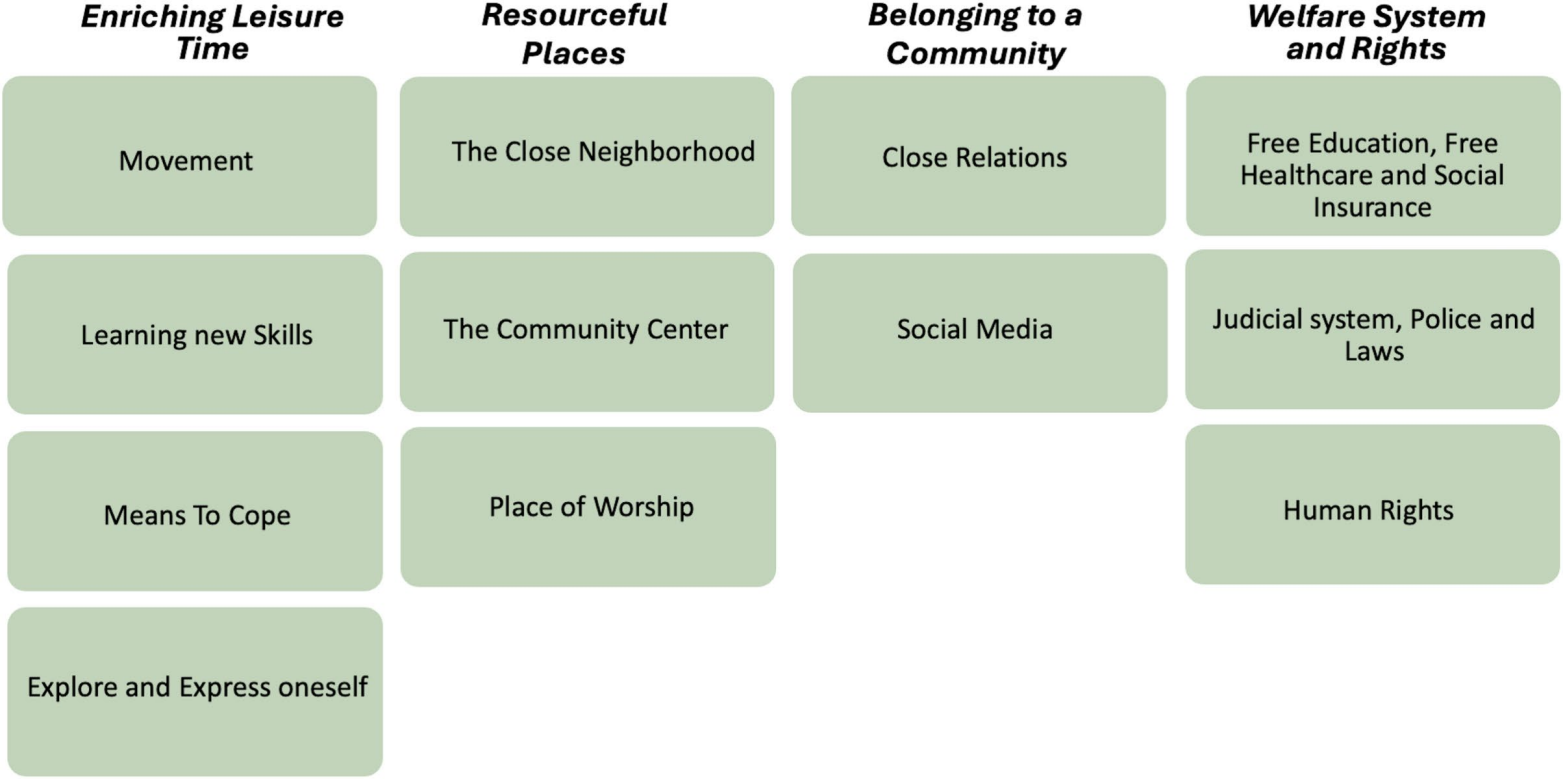
Visualizing main and sub-themes.

The analysis took the form of an inductive thematic analysis, inspired by the six-steps guide for analyzing data as described by Braun and Clark (64). The dialogs from the focus groups on factors related to well-being were recorded and transcribed by the first author to ensure that the coding process and thematization accurately followed the children’s words, capturing all perspectives and nuances. The coding process was conducted using the software program NVivo. Data analysis began with the first author familiarizing herself with the data, followed by the generation of initial codes, searching for themes, and then reviewing themes. This was an iterative process, which involved generating new themes and modifying some potential themes into sub-themes. Subsequently, the themes were named, and finally, the thematic analysis was written up. The final thematic map consists of four main themes with a total of 12 sub-themes (see Figure 5). The co-authors listened to the recordings and reviewed the transcripts. They also reviewed the preliminary sorting done by the first and second authors of the study, together with the children, through their well-being maps. Throughout the analysis process, a continuous dialog was maintained among all authors to ensure credibility.

### 2.3 Ethical considerations

The research was approved by the Swedish Ethical Review Authority (Reg. No. 2023-00979-01) and followed the Helsinki Declaration (65). Children and their guardians who signed up were given oral and written information about the study. The children were required to understand and speak Swedish, however, information to guardians was provided in Swedish, English, and Arabic. Guardians and children were informed that participation in the study was voluntary, and they could withdraw from the study at any time, without providing a reason, nor facing any consequences. Both children and their guardians provided their written informed consent ahead of the study start. During the research sessions, AAHs personnel were available to provide support to the children if sensitive discussions emerged in the group that could have made them uncomfortable. Communication with guardians were also maintained to ensure that the children were comfortable participating. An additional advantage of this study is that it was based in the AAH-premises, an environment familiar to the children.

## 3 Findings

The analysis resulted in four main themes, representing key factors related to child well-being: *Enriching Leisure Time, Resourceful Places, Belonging to a Community,* and *Welfare System and Rights*. According to the children, their immediate surroundings and close relations, as well as broader societal factors are important to their well-being. The children perceived these factors as intertwined, with structural issues affecting their personal and daily lives (see Figure 5) for a visualization of main and sub-themes.

### 3.1 Enriching leisure time

This theme, describing an Enriching Leisure Time as a factor related to well-being, was defined as fundamental by almost all children, and includes both physical and cultural activities. While the children enjoyed cultural activities in terms of singing, dancing and painting, they also expressed appreciation for culturally adapted activities, mentioning Easter celebrations, making gingerbread houses, but also celebrating Eid at the AAH. Additionally, the children highlighted that engaging in movement-based activities was beneficial for both body and mind, being energizing and enjoyable. Many emphasized the importance of having sports activities and breaks during the school day, as sitting still for too long made them feel tired.

*[Sport is important] because you must move your body a bit. If you don’t move, you feel tired and stuff like that. If you move, you feel energetic and good. (Girl, AAH2)*

According to the children, a pivotal aspect of doing activities was learning new skills. It was important as it improved their confidence, self-image, and made them feel good about themselves.

*My interests and hobbies make me happy. When you have a hobby, you feel that you have developed and become better at something. It’s fun to do something, having these hobbies and stuff. I like it. (Boy, AAH2)*

One specific thing that came up frequently when talking about well-being and self-development in relation to leisure time activities was “Friend of the Week.” During each activity at the AAH, leaders awarded the title “Friend of the Week” to a child who has demonstrated exceptional kindness, commitment, patience, helpfulness, or personal growth. This recognition was highly valued among the children, and receiving the award made them feel happy, proud and contributed to their well-being. Many noted how being named “Friend of the Week” helped them develop as a person, motivated them to improve and become kinder. They also felt proud to tell their parents about it which also positively influenced their well-being.

*I have developed a lot with the AllActivity House. With the AllActivity House, we have learned to be kinder, with 'The Friend of the Week' and stuff like that. (Boy, AAH2)*

Furthermore, some of the children described activities as a key factor to their well-being, as it was perceived as a form of refuge or means to cope and forget about their problems. Children described sports as an activity that transported them to a different world, where they could focus, have fun, and forget about everything else. Similarly, they perceived cultural activities, like listening to music, as an activity that gave them emotional support and possibilities to relax and reflect when they felt stressed, unfocused or sad.

*And I love music, and it’s so nice for me. When I’m a little sad, I put on music, and then I’m happy again. So, it calms me down. (Girl, AAH3)*

The children also described the importance of being able to explore and express themselves and who they are as individuals for their well-being. They talked about how activities and hobbies gave them an opportunity to do this. Various forms of artistic and creative expressions were identified as important for the children’s well-being, and crucial for them to build an identity for themselves:

*My hobbies… They are part of my identity, and what I think. (Girl, AAH1)*

### 3.2 Resourceful places

The second theme encompasses the importance of different places to the children, ranging from their close neighborhood, places for leisure time, as well as a place to practice religion. They emphasized the importance of feeling safe in their neighborhood, having good neighbors, and access to local community resources such as a nearby soccer field, playgrounds, nice courtyards and green spaces. They extensively discussed the relevance of keeping the neighborhood clean. In addition to being a place to play and socialize, the close neighborhood was described as a safe space for those needing to get away from conflicts at home.

*[Places outside are important] because you can be with friends outside. I'm not saying I have it, but maybe some don’t feel safe at home, or there are problems at home. Then you can be outside. If you go out, you get fresh air and do things that make you forget about it. Or you just think it’s fun to be out with your friends and do enjoyable things.* (Girl, AAH2)

The AAH was emphasized as an important place by the children, as it was associated with positive feelings, such as energy, safety, friendship, joy and community, but also access to material resources. They talked a lot about the importance of the activities offered at the AAH, the engagement among the leaders, and the material resources available, such as games, dance lessons, sports equipment and craft supplies. The children emphasized the value of the location of the AAH, being in the school-buildings, as this makes them accessible and easy to attend.

*I feel safe in the AllActivity House, and I have something to do. If I didn’t have the AllActivity House, I would just go home and sleep or check my phone.* (Boy, AAH3)

The importance of a place of worship was brought up by many of the children as a significant factor related to well-being. They appreciated having a specific place to practice their own religion. The children even perceived feeling safe, calm and content there. Many of the children described going to the mosque or church on a weekly basis and expressed the importance of a religious community which the place of worship provided.

*The mosque is important to me because when I go to the mosque and pray to God, I feel good. It feels like no one can touch me, or there won’t be fights or chaos and stuff like that.* (Girl, AAH3)

Additionally, many of the children expressed that their religion served as an important guiding star in an often changing and chaotic world. They strongly identified with their religion on a personal level and described their religion as an intrinsic part of themselves.

*My religion makes me safe. Because without my religion I don't know who I am. Without my religion I don't know what to do. And [without religion] there would just be a lot of people who didn't know what to do in life*. (Girl, AAH1)

### 3.3 Belonging to a community

The children described the fundamental role played by close relations to family and friends through the support and joy these relations provided. Social media was another important factor, as a tool to communicate, gain information and entertainment. However, the children also discussed problems related to social media, such as over-use and fear of being exploited.

The children spoke about the importance of feeling socially comfortable, safe, and experiencing a sense of belonging. Close relations were important in this regard, and family was described as central by all children. They spoke about their families in terms of happiness and love, emotional and material support, as well as safety.

*The family is important; we stick together and help each other. If someone gets hurt or sick, we help each other. And we have fun with each other, going out with the family and stuff.* (Girl, AAH3)

Parents and siblings were described as the most important individuals. However, relatives such as grandparents, aunts, uncles and cousins, and relations in their country of origin were also described as significant, often in terms of providing a social context, belonging and joy. The children expressed other relations in the local community as important for their well-being, and mentioned neighbors, friends and activity leaders. They described spending time with friends in and out of school, appreciated being themselves around friends, and perceived friends as a valuable support. The leaders at the community centers also played a pivotal role in the well-being of the children. They valued having a trusted adult outside of the traditional caregiver or teacher roles, someone more akin to a supportive friend. The children frequently expressed appreciation for the leaders’ kindness, humor, care, and overall support. Many reported having a close bond to and feeling a deep sense of trust in the leaders. They emphasized that they felt comfortable sharing personal thoughts without the fear of being judged.

*Because they have taught me how to do many things, and if I’m sad, they make me feel good and not be sad anymore.* (Girl, AAH1)

Social media was described as important for the children’s well-being, as it was a means to communicate with family, friends and relatives, but also to learn new things, receive information and news. Many of the children described having family and friends in other parts of the world, which made social media an essential communication tool to stay close and in contact with them. The children also described social media as valuable for keeping them informed about local and global news. However, through social media, they also received information about ongoing war and conflicts in other parts of the world, as well as criminal activities in their close neighborhood which affected their well-being.

*It [social media] helps me to know more about my neighborhood. I get information and I get to know things, like on YouTube or Snapchat. And, on social media, I get to talk to other people.* (Girl, AAH1)

While many expressed enjoying being on social media for entertainment, and learning for example how to cook, some problematized their use of social media. They described how it was easy to overuse their phones, and that excessive use negatively impacted their sleep and eyesight. The children also discussed instances of exploitation that had occurred through social media platforms, that worried them and made them feel unsafe on social media.

*I use them (social media platforms) for many things, it is good. But there are many things that are bad that happen here. Like people forcing people to maybe give them money, blackmail, or fights or quarrels. And then it gets really bad. And it only happens because we have social media.* (Girl AAH1)

### 3.4 Welfare system and rights

The fourth theme describes how the children self-perceived the importance of access to different welfare system functions, such as school and healthcare free of charge. They also highlighted the pivotal role of law enforcement and human rights for their well-being.

The children described the importance of living in a society where basic needs are met. They elaborated on the importance of free healthcare, free education, and good housing for their well-being. They also talked about the necessity of urban necessities, such as well-maintained walk and biking lines, public transport and access to grocery stores. Knowing that there is support and accessibility to necessities were described as important by the children in terms of feeling secure and happy in their everyday lives.

*The fact [that there is healthcare] makes me happier, and it doesn’t make me so afraid that something will happen because I can just go there [to the hospital] and they will help me.* (Girl, AAH2)

Through reflecting on financial aspects of social services, the children raised poverty-issues. They talked about the need for education, meals in school, and healthcare being free of charge. They problematized this by arguing that if school, for example, was not free of charge, people experiencing poverty would not be able to get an education. Furthermore, they would struggle in life by not finding a job and being able to provide for themselves and their families. Moreover, the children often reflected upon the need for financial support from the state to be able to access different societal services. One example is how they described the importance of social insurance from the state for families with unemployed parents, as this made it possible for the children to participate in society regardless of the financial situation at home.

*Imagine if you didn't have this, what is it called… the social insurance! What if they don't give money to the parents for the children, then the children couldn't have clothes to wear to school and all that.* (Girl, AAH2)

Going to school and obtaining an education were described as a vital factor related to well-being for the children. They stressed the importance of education in creating a good future for themselves, securing employment, and acquiring knowledge about the world. School was also seen as an important social environment where they could meet and spend time with friends, as well as attend the AAH.

*School is important because it helps me get a job and things like that. If we didn’t have school, I wouldn’t feel secure because then I wouldn’t get a job, I wouldn’t get food, I wouldn’t get those things. I also make friends, buddies, and everything like that. […] If school didn’t exist, I wouldn’t learn how to think, I wouldn’t have any friends.* (Girl, AAH1)

The general attitude toward school was positive, however, criticism was expressed regarding the lack of inclusion in decision-making, the length of lessons, insufficient adult-support, and a chaotic environment with a lot of conflicts.

*School is not fun. There are fights, there are people who say bad words. You don’t want to be called that.* (Boy, AAH3)

The children also described the need for police, laws, and courts to maintain order, prevent crime, and ensure safety. Many children explained that, without these institutions, chaos would ensue, leading to theft, fights, and other criminal activities. They viewed the judicial system as essential for feeling safe and secure in their daily lives.

*[…] if we didn’t have the police, it would feel like we couldn’t go out because we would think something might happen.* (Girl, AAH1)

Some children expressed doubts about the police and felt that they would not be able to protect them in emergencies, as it would take too long for the police to arrive if they were needed. The children also described feeling nervous around the police and expressed that it would be good if the police could be more present, and not just come during dispatch or emergency situations. They wanted to get to know the police to feel safer around them and build trust.

*The police don't make me safe. If someone comes running after me with a knife, I'm not going to use my time to get a police officer to help me.* (Girl, AAH1)

Human Rights was also identified as another key factor for well-being, with freedom of expression and religion specifically highlighted. While the children expressed the importance of having the right and freedom to believe in whatever they chose, they also emphasized the need to respect that people follow different religions, while others do not adhere to any religion. It seemed like the children thought, and talked a lot about freedom of religion, and freedom of expression, and that these were important issues to them.

*I can believe what I want, and I can go to church, and no one can influence me or force me to believe in something else.* (Girl, AAH1)

## 4 Discussion

**Figure 6:**
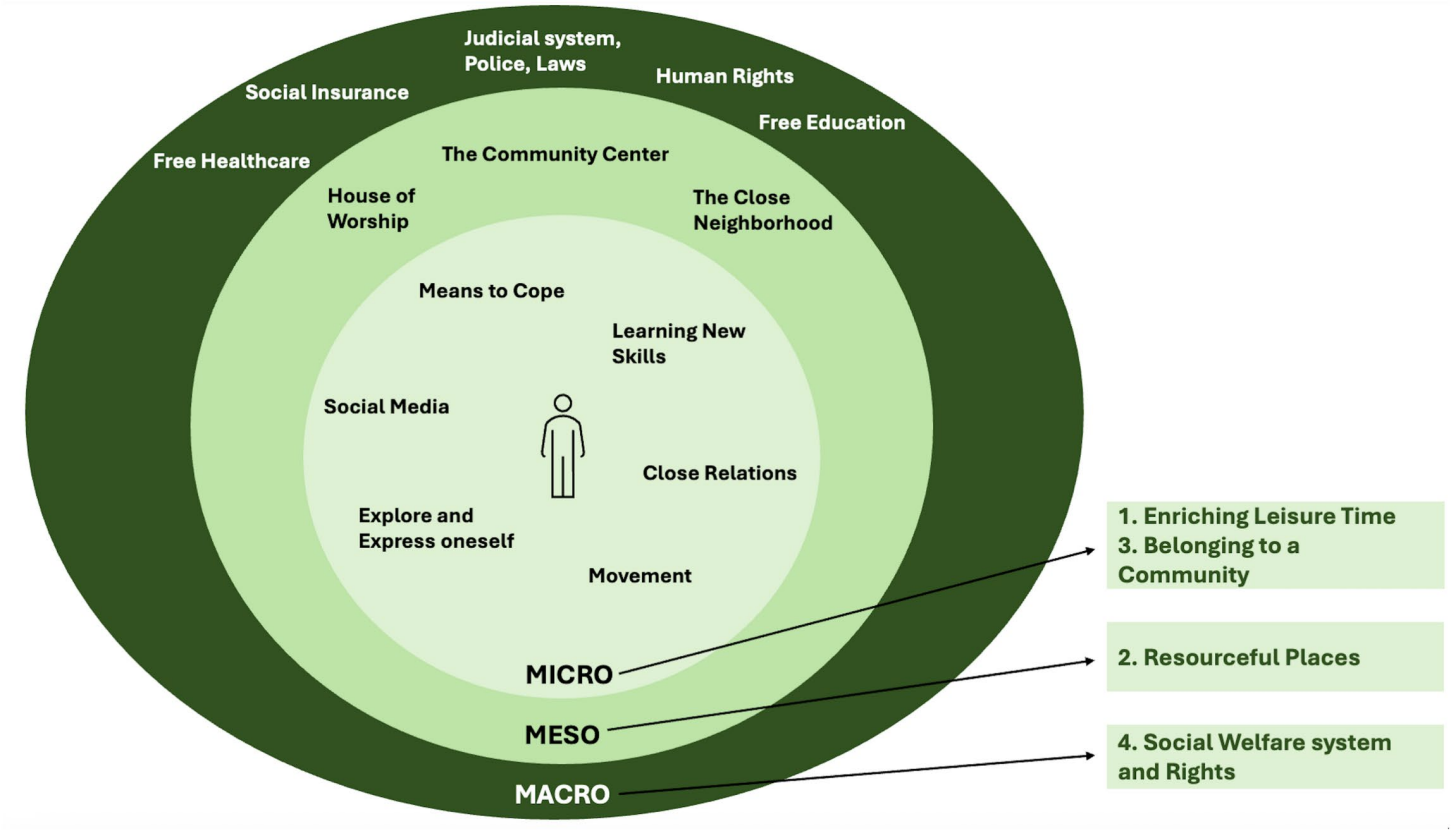
Results mapped across Micro, Meso and Macrosystem levels.

The aim of this study was to, through a participatory process, explore and enhance the understanding of key factors in the social context that contribute to child well-being among children with a migration background. The results provide a holistic understanding of child well-being, which is in line with the UNICEF definition (15), covering both structural and personal dimensions. Furthermore, the study supports Smith and colleagues’ view that socioecological models can deepen the understanding of child well-being, recognizing that broader social, physical, and environmental systems impact children’s possibilities to feel well (25). A simplified version of Bronfenbrenner’s ecological model of human development was used in this study, as the factors defined by the children extended across multiple levels in society related to the Micro, Meso, and Macrosystems (see Figure 6).

According to the results of this study, *Enriching Leisure Time*, meaning movement, learning new skills, means to cope and explore and express oneself, as well as *Belonging to a Community*, described as close relations and social media, relate to the microsystem. Further, the results of this study shows the importance of *Resourceful Places*, described by the children as the close neighborhood, community center and a place of worship, relating to the mesosystem. The result also shows that *Welfare system and Rights*, described by the children as education and healthcare free of charge, the judicial system, laws and police, as well as human rights, are crucial to child well-being, and adhere to the macrosystem (see Figure 6).

The children’s descriptions about having an *Enriching Leisure Time*, is in line with previous research by McAuley (66), describing that learning new skills through participation in different activities is crucial for child well-being. This also aligns with Capurso and Pazzagli’s finding that play, although often overlooked, is crucial for building resilience, improving emotional well-being, regulating emotions, and promoting problem-solving flexibility (67).

In our study, the children perceived activities as a diversion from the perpetual difficulties in their everyday life. They described artistic expressions, such as dancing, painting, and creating music, as particularly effective in helping them explore and express themselves, positively impacting their well-being. This has been seen in previous research (68, 69), and aligns with findings from a systematic review conducted by Benninger and Savahl, describing that children’s ability to construct a sense of self is a key to their well-being (70). Self-identity is shaped by roles, behaviors, and children’s personal experiences in their environment, and has also been shown to evolve over time as individuals encounter new experiences and environments, influencing their sense of individuality and distinctiveness from others (71, 72). The children participating in this study often moved between different geographical contexts, such as their country of origin and their host country, learning to navigate the mixture of influences and expectations they had to manage. As described by Benninger and Savahl (70) and Isom (73), children often feel the need to negotiate their cultural identity and position themselves in relation to the majority norms and expectations in the society.

Creating a sense of self is part of *Belonging to a Community*, encompassing close relations and social media, described as important factors to child well-being in this study. These factors adhere to the Microsystem in Bronfenbrenner’s ecological theory of human development (40), as they relate to the children’s direct environment and daily interactions. In terms of close relations, the children described their family, relatives, friends, as well as another trusted adult, such as an activity leader, as pivotal for their well-being. Previous studies have also shown that well-functioning family and peer relations, as well as, support from a trusted adult during childhood, significantly contribute to well-being in both childhood and into adulthood, particularly among those who experience significant early-life adversity (74, 75). The children in this study seem to elaborate on *Social Cohesion*, a concept including three essential features: social relations, connectedness, and an orientation toward the common good (76–78). Previous research by McDonell and Sianko found that children in socially cohesive neighborhoods report higher life satisfaction. These children may also experience more freedom and independence, as their parents feel safe allowing them to be outdoors in the neighborhood on their own (79). Oh et al. further argue that social cohesion can mitigate the risk of mental health issues and depression in children and adolescents growing up with material hardship (80), which relates to this study, conducted in areas with high child poverty. Related to social relations and connectedness, the children described social media as a crucial tool to communicate with family, relatives and friends, locally as well as in other parts of the world. Previous research has also shown that children use social media for similar reasons (11, 81, 82). However, to our knowledge, studies concerning this use of social media among children below the age of 12 seem sparse.

The factors identified by the children that adhere to the Microsystem interact with their descriptions of other key factors for well-being, namely *Resourceful Places*, including the close neighborhood, the community center, and a place of worship. These factors relate to the Mesosystem in that they are forums for interaction between different microsystems. As argued by Jack, the social relations that are pivotal for children’s well-being do not occur in a vacuum (83), and the different factors the children in this study described as crucial to their well-being were tied to *Place*. This has been shown in previous research about place-identity, which refers to parts of a person’s self-concept shaped by interactions with, and meanings attached to physical environments (84, 85). The children described how different places in their community facilitated a platform for them to be active, socialize, develop as people, feel happy, comfortable, and safe in their everyday life. This has also been shown by Eriksson, who emphasized the importance of places that foster togetherness, activities, and positive emotions for children’s well-being (86). The children in our study further expressed a personal identification with their place of worship and elaborated on the importance of having a space to practice their religion and be part of a religious community. This is in line with previous research, describing religion as an important component for children’s well-being, offering a community, self-esteem, and purpose in life (87, 88).

The importance of a community center, such as the AAH, was also described as a key factor related to child well-being in terms of socialization, and activities. This aligns with another recent participatory research study by Enskär et al. (89). Interestingly, the children in our study further emphasized their appreciation of being involved in different forms of decision-making at the AHH, for example regarding which activities should be provided.

The children in our study described factors related to well-being in terms of the Welfare System, Judicial System, and Human Rights, which correspond to the Macrosystem in Bronfenbrenner’s ecological theory (40), as they reflected on broader societal norms, institutions, and structural issues. Through their dialogs, the children elaborated both on the importance of having their basic needs met, like access to adequate housing, education and healthcare free of charge, but also discussed access to other material needs. Previous research show how low socio-economic status adversely affects children’s physical and mental health and well-being, as well as cognitive development (90, 91), with effects up in adulthood (92). Baltica Cabieses and Wilkinson (90) have showed that income inequality and child poverty negatively affect children’s well-being even in wealthy countries. Our study was carried out in disadvantaged areas with high levels of child poverty (20, 58), and the results indicate that many basic needs might not be guaranteed within the family due to financial hardship. As emphasized by Hajime et al., and Cabieses and Wilkinson, this study highlights the need for strengthened compensatory measures to ensure that children’s needs are met and protected, in order to support child well-being (90, 93).

Through participatory dialogs and reflections spanning over a longer period of time, the children in our study wavered between different aspects related to well-being. They described ambivalence and often raised both positive and negative aspects regarding the same topic, such as the school, social media and police. Although attending school was described as fundamental by most of the children, many described the school to be a chaotic environment without sufficient adult-support. Similar findings are shown in a research by Opara et al. (94) who describe that insufficient resources, large class sizes and an unfavorable environment, contribute to a more strained learning climate. Further, the children in our study perceived social media platforms as important channels for communication, yet also described them as an unsafe environment posing many risks, a result consistent with previous research (95). The police were discussed as very important for the children’s sense of security in our study. However, their perceptions were not wholly positive as they described that they often felt nervous around the police, and wished for better relations so they could build trust toward them. This aligns with earlier research, indicating that children with migration background tend to have lower confidence in the police compared to other groups (96, 97). While most existing research has focused on adolescents, Fine et al. (96) demonstrates that trust in the police among younger children, aged 10–11, can significantly improve through positive, non-repressive interactions between children and police officers, for example through community-based programs.

Based on the children’s dialogs, Capacity Building in the close neighborhood seems essential to child well-being. While varying in meaning, capacity building is well acknowledged in health promotion, and WHO has defined it as the improvement of “both the ability of individuals to take action, and the capacity of groups, organizations or communities to influence the determinants of health” (98). In our study, the value of working with community health promotion building capacity in the community becomes evident through the various key factors related to child well-being that were identified. Such efforts should prioritize children’s access to well-functioning institutions, opportunities to build a sense of belonging, and close relationships with both adults and peers. Further, as this study shows, it is crucial that children are able to engage in enriching leisure activities, where they can develop as individuals and explore their self-identity. Promoting child well-being therefore requires places where children, through participatory processes, gain ownership and can achieve empowerment.

## 5 Limitations and strengths

This study was conducted together with children from three socioeconomically disadvantaged geographical areas in Malmö. As all AAHs are situated in urban areas, children living in rural settings were not part of this study. Also, since only children attending the AAH were included, children who did not attend the AAH for various reasons are not represented, which limits the representativity of the study. However, the children who participated had diverse backgrounds, were of mixed genders, and ranged in age from 9 to 12 years old, enhancing the study’s representativity. The children in this study themselves mentioned that they did not perceive the age difference between them as a challenge during the sessions. The younger children articulated their personal perceptions and did not seem to want to fit in or mimic the older children’s responses. Many even expressed that it was enjoyable to participate in activities with children from different age groups.

Conducting research with children, especially qualitative research, requires extra caution due to numerous ethical challenges, ranging from obtaining informed consent, bias, and the children’s integrity, all related to power imbalance between the child participants and the adult researchers (99–101). However, guided by Freire’s critical pedagogy, the research process of this study has been designed to minimize hierarchies and power imbalances through close collaboration and continuous dialog with the children, emphasizing equality and the importance of their expertise. Decisions regarding research activities have been made together within the CBPR-teams, and an environment of openness and critical thinking has been supported so the children could freely engage in dialog without fearing for consequences. Although the children did not initiate the project, they discussed and agreed on the research question and aims. They recognized the importance of the study and contributed their perspectives, ensuring the goals were meaningful to them. Subsequently, the research activities were designed to suit the children and were approved by the children themselves. For example, the photo-voice method used, is considered a child-friendly approach that empowers children to express their perspectives and actively participate on their own terms, controlling and shaping the conversations through the images they take (61, 62).

Well-being is a complex concept for the children to understand and discuss and this could have limited in-depth exploration. To support them to grasp and explore this concept, prolonged engagement was necessary. Although the topic was perceived as complex initially, through various activities and ongoing dialog, the children’s reasoning and analytical participation developed over time. The research also began with a trust-building phase where the CBPR-team got to know each other through playful activities. This phase was crucial for establishing strong relationships and trust. Weekly planning-meetings between the first and second authors and the activity leaders allowed for the adaptation of activities based on the needs of different child groups. This close relationship with the activity leaders strengthened the process, facilitated strong engagement and trust between the researchers and children in the CBPR-teams, thereby creating a comfortable space for dialog.

## 6 Conclusion

The current study explored key factors related to child well-being, from children’s own perspective. Four main themes were identified: Enriching Leisure Time, Resourceful Places, Belonging to a Community and Welfare System and Rights. All these findings could be related to Bronfenbrenner’s socioecological model of human development. Based upon the main findings, community health promotion working toward community capacity-building is key to support child well-being. Such initiatives should consider children’s access to well-functioning institutions, like quality education where they thrive and develop, but also access to well-functioning judicial systems and human rights. Furthermore, the work should focus on strengthening places defined as important for child well-being. For example, by creating neighborhoods that support children’s opportunities to build a sense of belonging, and close relationships with both adults and peers, as well as access to enriching leisure activities. Since the result of this study show the importance of children’s ability to learn new skills and explore their self-identity, child well-being also requires participatory processes, supporting children to achieve empowerment. Further research should focus on developing strategies that are contextually relevant and collaborate with key stakeholders to support and ensure children’s access to the factors they perceive and define as pivotal to their well-being. The research team conducting this study will continue to work within the project and focus on actions.

Lastly, this study demonstrates the importance of a strength-based approach, ensuring children’s participation in all efforts aimed at their well-being, and supporting ownership among children.

## Data Availability

The original contributions presented in the study are included in the article/supplementary material. Further inquiries can be directed to the corresponding author/s.
